# Diverse new plasmid structures and antimicrobial resistance in strains isolated from perianal abscess patients

**DOI:** 10.3389/fmicb.2024.1452795

**Published:** 2024-10-15

**Authors:** Zhen Xu, Lulu Shi, Tao Meng, Mei Luo, Jiaming Zhu, Mingyu Wang, Wenlong Shen

**Affiliations:** ^1^Department of Anorectal Surgery, Qilu Hospital (Qingdao), Cheeloo College of Medicine, Shandong University, Qingdao, China; ^2^State Key Laboratory of Microbial Technology, Microbial Technology Institute, Shandong University, Qingdao, China; ^3^The Affiliated Hospital of Qingdao University, The First Clinical Medical College of Qingdao University, Qingdao, China; ^4^School of Life Sciences, Shandong University, Qingdao, China

**Keywords:** plasmid, Nanopore sequencing, antimicrobial resistance, perianal abscess, mcr-10.1

## Abstract

**Introduction:**

Plasmids, the most important and versatile bacterial extrachromosomal DNA Molecules, has have been a center central topic for bacterial genetics and biology. However, the inability of short-read high-throughput sequencing methods to reliably assemble plasmids makes it difficult to investigate the diversity of plasmid structures and functions.

**Methods:**

In this work, we used the long-read Nanopore sequencing method to address this issue, by producing high quality whole genome sequences of 33 bacterial strains from 11 perianal abscess-suffering patients.

**Results and discussion:**

Successful high quality assemblies were generated with this method, including 20 perfect assemblies out of 33 genomes. A total of 47 plasmids were identified from the bacterial strains, including 12 unique, newly identified, high quality circular plasmids. These plasmids were further subject to structural analysis, leading to the finding of significant diversification from previously known plasmids, suggesting the diversity of plasmid structure and function. Particularly, two *mcr-10.1*-harboring conjugative plasmids were found from *Citrobacter portucalensis* and *Enterobacter kobei*, which were not previously reported. This works shows the feasibility of using long-read sequencing to identify plasmids, and the high diversity of plasmid structure and function that awaits further surveillance.

## 1 Introduction

The central dogma of modern biology indicates that most biological functions are encoded in genomes that are either DNA- or RNA-based (Song, 2021). Deciphering these codes for biological functions has been the most important task of modern genetics and physiology. This is been the primary motivation for initiating and succesfully finishing one of the most important scientific projects of our times—the Human Genome Project (Green et al., 2015). The completion of this project in 2003 has started a new era of biology, where sequencing of genomes has become the key biotechnology and centerpiece for biological research.

The enormous success of high-throughput sequencing technologies in the past 20 years has vastly deepened our understanding on life forms and functions. This does not mean it is perfect. The 2nd generation sequencing that is dominating the world of biological research only produces short reads in the range of 100–400 bp, which led to tremendous difficulties in stiching (or assembling) them together to generate the whole genome sequence (Hu et al., 2021). Commonly, dozens or hundreds of fragments (or contigs) are generally produced for each chromosome, although biologically it should’ve been only one piece. This is not a real problem for larger, eukaryotic genomes, as genes are relatively sparse on the genome, and having a few gaps do not normally matter.

For bacteria, it is a totally different story. Besides the main chromosome, bacteria normally often carry extrachromosomal circular DNA molecules, the plasmids, for additional functions that are not encoded on the chromosome (Carroll and Wong, 2018). The plasmids are the variable parts of bacterial genome, which bring quick adaptation, high versatility, and beneficial traits bacteria needed for combating changing environment with a small genome. Unlike selection for new functions that normally take a very long time, plasmids can be replicated and transferred between bacterial cells leading to very quick acquisition of functions (Partridge et al., 2018). This is essential for surival under stresses such as antibiotic treatment, while acquisition of antibiotic resistance genes (ARGs) hosted on plasmids is a primary route for antimicrobial resistance (AMR) by bacteria (Li et al., 2018).

The short reads of 2nd generation sequencing methods make it very difficult to detect, let alone assemble plasmids (Arredondo-Alonso et al., 2017). Therefore, our knowledge on the plasmids, including their sequence variabilities, the diversity of plasmid functions, has been limited. The recent emergence of long-read 3rd generation sequencing methods that can produce reads of up to several mega basepairs showed very good promises in solving this problem (Loman et al., 2015), as they can, sometimes, sequence the whole plasmid (normally <200 kb in size) in one read.

This work aims to understand how much we are falling behind in understanding the plasmid diversity and function, and how we can address this issue with improved 3rd generation sequencing technologies. This was done by attempting to determine plasmids from bacterial strains from isolated clinical samples, checking to see how many plasmids have not been previously observed, and identifying new plasmid structures and functions. Samples from perianal abscess-suffering patients were used, because this is a disease caused by bacterial infection (Sahnan et al., 2017), and commonly applied antibiotic theapy might result in stress leading to enriched plasmid occurrence (Brar et al., 2020; Ghahramani et al., 2017; Lohsiriwat et al., 2010; Mocanu et al., 2019). It is also a very common disease that represents a large proportion of humans (Afşarlar et al., 2011). The studies undertaken here provides a surveilience of a small but conclusive cohort, leading to the conclusion that plasmid diversity on structure and function is still under-investigated, and additional surveillance efforts with 3rd generation sequencing need to be made.

## 2 Materials and methods

### 2.1 Sample collection

Samples of 11 patients with perianal abscess were collected from March to November, 2023 in Qilu Hospital (Qingdao) of Shandong University. For each patient, a sterile swab was used to swab three locations (anal skin, feces, and abscess) and then stored in sterile 10% glycerol solution and transported to the laboratory at low temperature (4°C).

### 2.2 DNA extraction, library preparation and sequencing

LB plates were used to culture bacterial strains from each sample. A single colony was randomly selected from each LB plate for sequencing. Total DNA was extracted using bacterial genome DNA extraction kit [Tiangen biochemical technology (Beijing) Co., Ltd.] according to the manufacturer’s instructions. Libraries for Nanopore sequencing were prepared using Rapid Barcoding Kit 96 V14 (SQK-RBK114.96) according to protocols. Nanopore P2solo with R10.4.1 flow cell was used for sequencing.

### 2.3 Data analysis

Basecalling and sequence demux was done by Dorado version 0.5.3.^1^ Sequence quality was evaluated and filtered using software package NanoQC version 0.9.4 and Chopper version 0.7.0 (De Coster and Rademakers, 2023). Whole genome sequences were assembled and corrected using software package Flye version 2.8.1-b1676 and Medaka version 1.12.0 (Kolmogorov et al., 2019). Flye was also used to determine sequence circularity. The assembled sequences were evaluated using software packages Busco version 5.2.2, Checkm2 version 1.0.1 and Quast version 5.0.2 (Manni et al., 2021; Mikheenko et al., 2018; Parks et al., 2015). Taxonomy was determined using GTDB-Tk version 2.1.1 (Chaumeil et al., 2022) AMRfinder version 3.11.26 was used to identify ARGs (Feldgarden et al., 2021). Plasmidfinder version 2.1.1 was used for typing plasmids (Carattoli and Hasman, 2020). Genome annotation was performed with Prokaryotic Genome Annotation Pipeline or Prokka version 1.14.6 (Li et al., 2021; Seemann, 2014). Snippy version 4.6.0^2^ was used to calculate the number of single nucleotide polymorphisms (SNPs). Putative plasmid sequences were searched against the NT database with the Blastn algorithm embedded in Blast version 2.15.0+ (Camacho et al., 2009). Plasmid copy numbers were calculated by dividing the sequencing coverage of chromosomes by sequencing coverage of plasmids. SnapGene version 7.2.1 and EasyFig version 2.2.5 were used for plasmid visualization and comparison (Sullivan et al., 2011).

## 3 Results and discussion

### 3.1 Patient and isolated strains

Samples were taken from 11 patients who were suffering from peranal abscess and received surgery at Qilu Hospital (Qingdao). One bacterial strain was isolated from anal skin, feces, and abcess from each patient, with a total number of 33 strains. The strains were subject to Nanopore whole genome sequence analysis, and had their taxonomy determined to the species level with GTDB-tk. The information on the patients and isolated bacterial strains can be found in Table 1.

**Table 1:** Information on patients and isolated bacteria.

Patient	Gender	Age	Strain	Isolated location	Taxonomy	Number of contigs
N6	Female	22	N6-1	Perianal skin	*Enterococcus faecalis*	4
			N6-2	Feces	*Enterococcus faecalis*	2
			N6-3	Abscess	*Enterococcus faecalis*	2
N59	Female	51	N59-1	Perianal skin	*Enterobacter kobei*	9
			N59-2	Feces	*Citrobacter portucalensis*	2
			N59-3	Abscess	*Escherichia coli*	3
N98	Female	25	N98-1	Perianal skin	*Klebsiella quasipneumoniae*	1
			N98-2	Feces	*Klebsiella variicola*	2
			N98-3	Abscess	*Escherichia coli*	2
N112	Female	32	N112-1	Perianal skin	*Staphylococcus aureus*	3
			N112-2	Feces	*Escherichia coli*	6
			N112-3	Abscess	*Staphylococcus aureus*	2
N183	Female	42	N183-1	Perianal skin	*Klebsiella pneumoniae*	2
			N183-2	Feces	*Escherichia coli*	7
			N183-3	Abscess	*Escherichia coli*	3
N119	Male	64	N119-1	Perianal skin	*Klebsiella pneumoniae*	2
			N119-2	Feces	*Escherichia coli*	3
			N119-3	Abscess	*Klebsiella pneumoniae*	2
N129	Male	21	N129-1	Perianal skin	*Proteus mirabilis*	1
			N129-2	Feces	*Proteus mirabilis*	1
			N129-3	Abscess	*Escherichia coli*	3
N157	Male	56	N157-1	Perianal skin	*Klebsiella pneumoniae*	4
			N157-2	Feces	*Escherichia coli*	3
			N157-3	Abscess	*Klebsiella pneumoniae*	3
N176	Male	34	N176-1	Perianal skin	*Enterobacter hormaechei*	4
			N176-2	Feces	*Enterobacter hormaechei*	1
			N176-3	Abscess	*Enterobacter hormaechei*	2
N193	Male	54	N193-1	Perianal skin	*Escherichia coli*	3
			N193-2	Feces	*Escherichia coli*	6
			N193-3	Abscess	*Escherichia coli*	8
N211	Male	41	N211-1	Perianal skin	*Klebsiella pneumoniae*	2
			N211-2	Feces	*Klebsiella pneumoniae*	2
			N211-3	Abscess	*Escherichia coli*	4

The isolated 33 strains belong to 10 species in seven genera. The majority of the strains (28) are Gram negative bacteria, all of which belong to the Enterobacteriaceae family. The isolated strains include 12 *E. coli*, seven *K. penumoniae*, three *E. hormaechei*, three *E. faecalis*, two *S. aureus*, two *P. mirabilis*, one *E. kobei*, one *C. portucalensis*, one *K. quasipneumoniae*, and one *K.* var*iicola* strains. All isolated bacterial strains are known opportunisitic pathogens, and all isolated *Enterobacter* strains belong to the *Enterobacter cloacae* complex.

With 3rd generation Nanopore sequencing, we were able to obtain high quality whole genome sequences for all the isolated bacterial strains, with an average of 3.15 contigs assembled per bacterium (Supplementary Table S1). Perfect genome assemblies in which all DNA molecules were assembled to circular form were obtained for 20 of the 33 bacteria. Of all 104 assembled contigs, 76 (73.1%) are circlar. These statistics confirm the high quality of the genome sequences and assemblies.

### 3.2 Plasmid diversity of isolated strains

**Figure 1:**
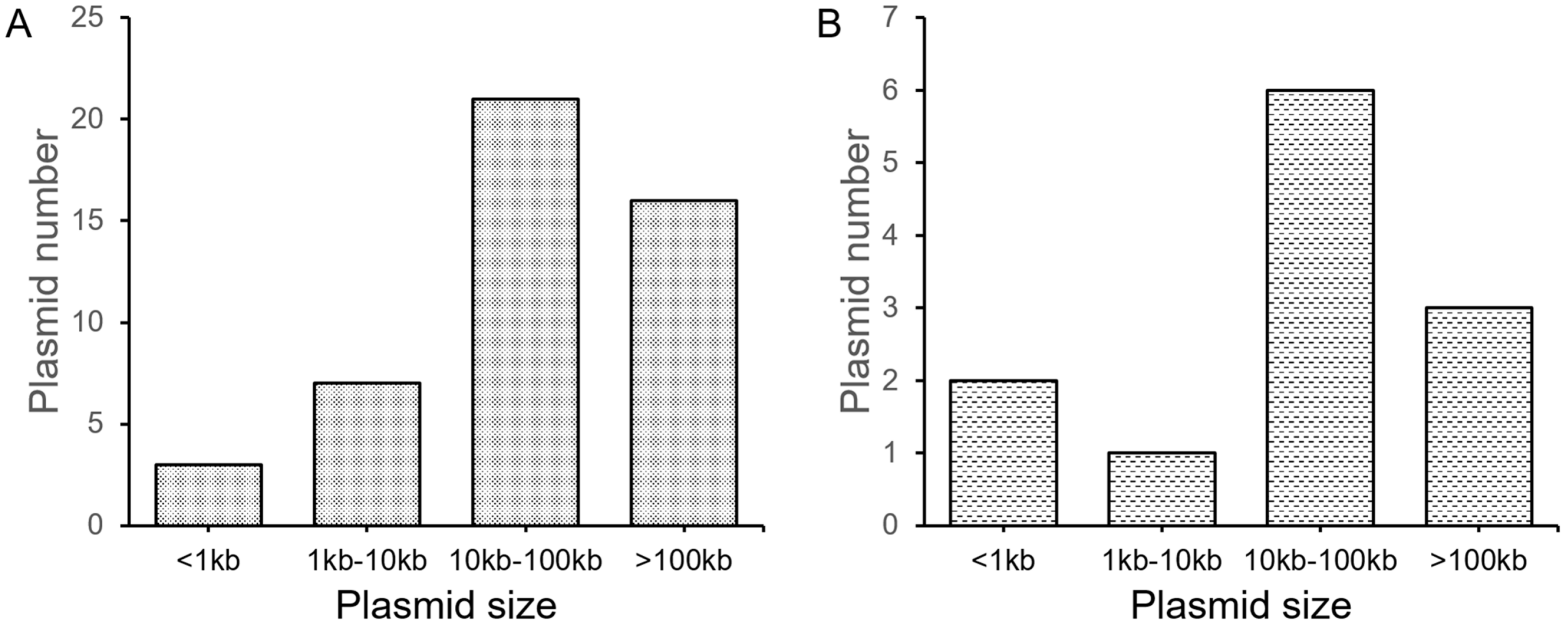
Distribution of plasmid sizes. **(A)** all plasmids; **(B)** unique new plasmids.

Of the 33 strains isolated in this work, only five (one *K. quasipneumoniae*, two *P. mirabilis*, one *E. hormaechei*, and one *E. coli* strains) do not harbor plasmids (Supplementary Table S1), suggesting the high prevalence of plasmids in human-related bacteria. On average, each strain contains 1.68 plasmids. Most plasmids have a size of over 10 k bp (Figure 1A), with an average size of 80.89 kb. It needs to be noted that very small plasmids (< 1 kb, 3 counts) also exist. Their existence was determined by circularity of sequenced DNA molecules, the presence of protein-coding genes, as well as their generally high copy numbers. However, circular DNA can also indicated excised products by recombinases *in vivo*. Therefore, the prediction of these very small “plasmids” may not be accurate. Also, 12 small contigs in five strains were assembled, which are part of a plasmid. We suspect there are still more plasmids that have not been fully assembled, although we believe most of the plasmids have been recovered with the method undertaken.

Of the 47 plasmids found, 41 are unique plasmids, of which incombatibility groups were determined for 31 plasmids. As can be seen from Supplementary Table S1, most of the plasmids (64.52%) are IncF plasmids. IncF plasmids are Enterobacteriacea-only plasmids that often have many replicons (like the IncFIB/IncFII plasmids found in this work) (Rozwandowicz et al., 2018). It is also the most frequently observed plasmid incompatibility group of human and animal origin (Rozwandowicz et al., 2018).

In attempt to identify new plasmids arising from the isolated strains, the Blastn program was used to search for similar DNA in the NT database. To classify a plasmid as a previously found plasmid, a known plasmid has to be present in the NT database that is 80–125% in size with the analyzed plasmid, the coverage-per-subject needs to be over 80%, and all High-Scoring Segment Pairs (HSPs) need to be highly similar (sequence identity >90%). Putative new plasmids were manually compared with the closest plasmid in the NT database to confirm their novelty.

A total of 12 new plasmids were found, which is quite a surprise. The distribution of new plasmid sizes is close to all plasmids found, with an average size of 58.80 kb (Figure 1B). All of the 12 new plasmids were assembled to circularity, confirming the quality of sequences. These new plasmids include four high-copy (predicted copy number > 10) plasmids and eight low-copy (predicted copy number < 10) plasmids.

The large number of new plasmids found in this work suggests the lack of our knowledge on plasmids. Plasmids are the most variable sections of the genome, and the lack of knowledge on plasmids reflects technological limits on plasmid identification, most likely due to the inability of commonly-used 2nd generation high-throughput sequencing methods to efficiently identify plasmids, due to their short reads. The 3rd generation long-read Nanopore sequencing used in this work solved this issue, and can generate high quality whole genome sequences as obtained in this work. Therefore, we were able to successfully identify all DNA sequences inside cells, including plasmids or integrons there were difficult to identiy with 3rd generation long-read sequencing methods.

**Figure 2:**
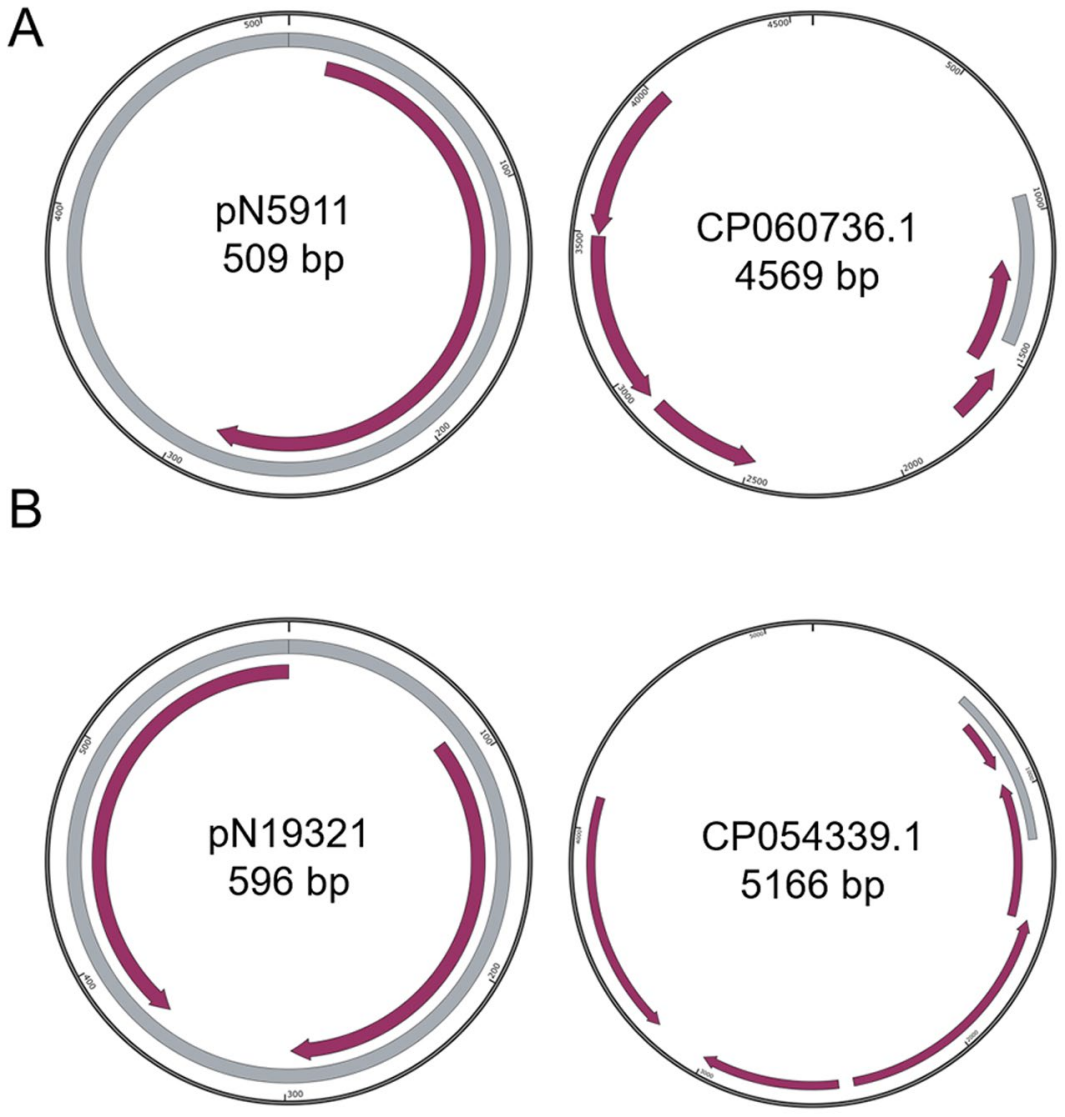
Comparison of small plasmids with closest known plasmids. **(A)** pN5911; **(B)** pN19321. Grey color indicates homologous regions. Purple arrows indicate coding regions.

**Figure 3:**
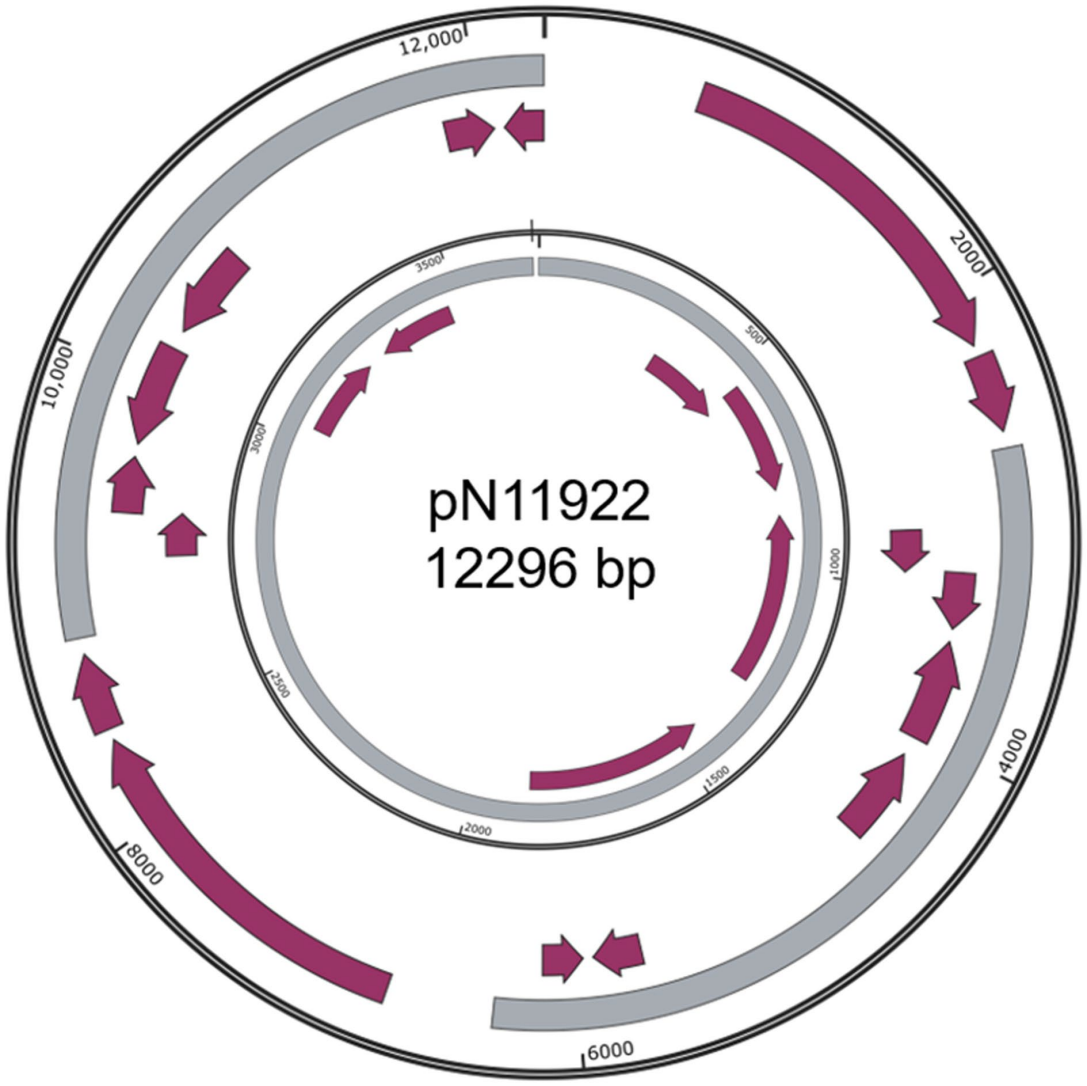
Structure of pN11922. Outer ring, pN11922; Inner ring, pHNBYF36-3. Grey color indicates homologous regions. Purple arrows indicate coding regions.

### 3.3 Structures of newly identified plasmids

The structures of newly identified plasmids were analyzed, and compared with their most similar counterparts found in the NT database.

Besides the three simpler and somewhat dubious plasmids: pN5911 (509 bp) from *E. kobei* N59-1 (Figure 2A), pN19321 (596 bp) from *E. coli* Z193-2 (Figure 2B) and pN11922 form *E. coli* N119-2 (Figure 3), all the other nine plasmids are clearly new, functional, and replicative plasmids.All of them carry replication initiation proteins (Rep proteins), and the majority of them are mobile plasmids. They are at least 20% different from similar plasmids, making them new plasmids. In addition, they also encode important physiological functions. All the annotation files are provided in the supplementary materials.

**Figure 4:**
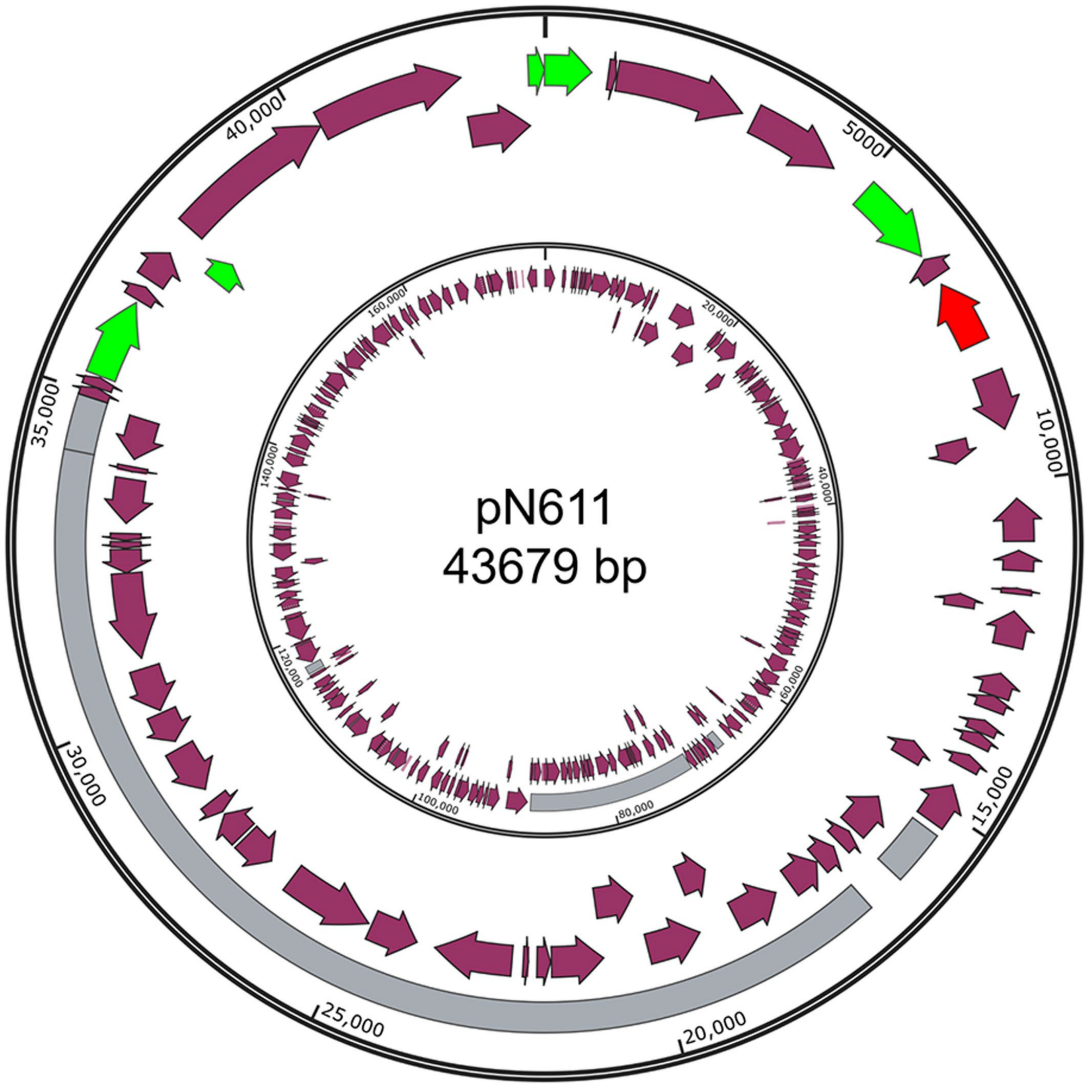
Structure of pN611. Outer ring, pN611; Inner ring, pT17-1-1. Grey region indicates similar regions. Green color indicates mobility related genes. Red color indicates the *rep* gene. Purple arrows indicate coding regions.

Only one of these new plasmids was from Gram positive bacteria: pN611 from *E. faecalis* strains N6-1 (Figure 4). The closest plasmid to pN611 is pT17-1-1 from *E. faecium* T17-1 (Genbank accession CP109841.1) (Chen et al., 2024). Comparison between the two plasmids showed that about half of pN611 originated from pT17-1-1, but the other half has no similar regions in this plasmid (Coverage: 44%, Identity: 99.67%). It is interesting to see that mobility related genes and the replication initiator gene on pN611 are located on the half that is not related to pT17-1-1. Considering replication and mobilization are the most important functions of plasmids, we suspect that pN611 is a result of a recombination event, leading to the uptake of part of pT17-1-1 into a plasmid backbone. The unique region of pN611 harbors tetracycline-resistance genes *tetM* and *tetL*, as well as a bacteriocin biosynthesis gene cluster, granting pN611 additional AMR and bactericidal features.

All remaining newly identified plasmids are from Enterobacteriaceae. Five of them are smaller sized plasmids (smaller than 100 kb). These plasmids include pN17631 from *E. hormaechei* N176-3, pN11921 from *E. coli* N11921, pN18311 from *K. pneumoniae* N183-1, as well as pN19332 and pN19335 from *E. coli* N193-3.

**Figure 5:**
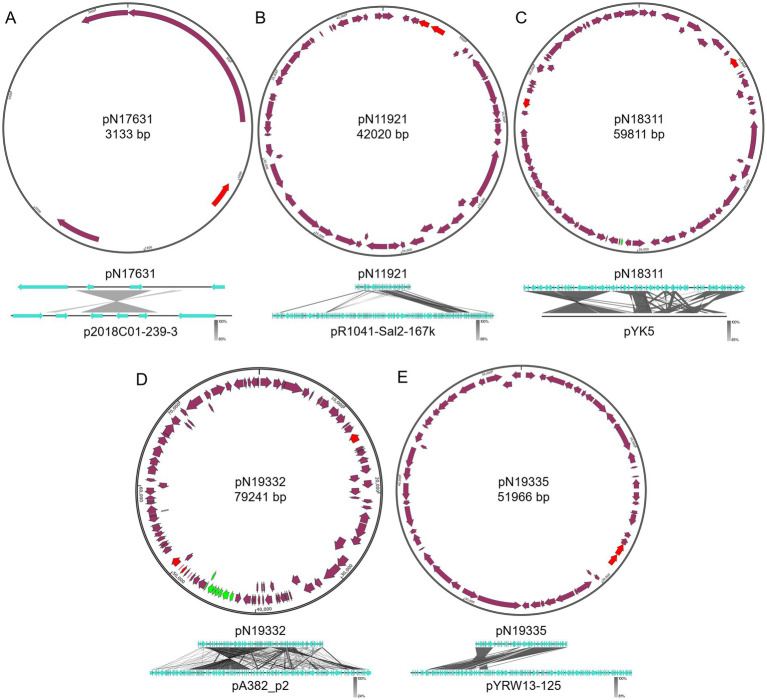
Structures of pN17631, pN11921, pN18311, pN19332, and pN19335. **(A)** pN19631; **(B)** pN11921; **(C)** pN18311; **(D)** pN19332; **(E)** pN19335. Green color indicates mobility related genes. Red color indicates the *rep* gene. Purple arrows indicate coding regions.

pN17631 has the closest homology to p2018C01–239-3 from *K. pneumoniae* 2018C01-239 (Genbank accession CP044388.1, Figure 5A).^3^ Besides sharing an antitoxin VbhA family protein-coding gene and a *rep* gene, pN17631 encodes an additional Abi protein and a hypothetical protein. p2018C01–239-3 encodes two additional hypothetical proteins and a helix-turn-helix protein, making them two distinct plasmids (Coverage: 41%, Identity: 88.46%).

pN11921 is an IncX1 plasmid that has the closest homology to pR1041-Sal2-167 k (Genbank accession OR095745.1, Figure 5B)^4^ from *Salmonella enterica* R10-SAl2 (Coverage: 46%, Identity: 99.97%). About half of pN11921 shares high sequence homology with pR1041-Sal2-167 k. However, the other half encodes key plasmid functions including replication initiation (*rep*) and segratation (*par* genes). pN11921 contains two additional ARGs, the aminoglycoside resistance gene *aph(3′)-Ia* and the *β*-lactam resistance gene *bla*_TEM-1_. Therefore, we believe pN11921 has a different plasmid backbone, and incorporates a fragment of pR1041-Sal2-167 k.

pN18311 is a IncFIA/IncR hybrid plasmid that has the closest homology to pYK5 from *Klebsiella* sp. TR5 (Genbank accession MK648236.1) (Yin et al., 2020) (Coverage: 77%, Identity: 99.99%). Besides a *rep* gene shared between pN18311 and pYK5, pN18311 contains a second *rep* gene. It is therefore suggested that pN18311 is a hybrid plasmid with a recombination event between two types of plasmids. It can also be seen with the comparison between pN18311 and pYK5 that quite complex recombination events took place even between the homologous regions of the two plasmids (Figure 5C). pN18311 also carries tetracycline resistance gene *tetD* and chloramphenicol resistance gene *floR* that were not part of pYK5.

*E. coli* N193-3 carries two new plasmids, a IncFIB/IncFIC pN19332 plasmid that shows the highest homology with pA382_p2 (Genbank accession CP142551.1) (X. Li et al., 2024) from *E. coli* ExPEC_A382 (Coverage: 72%, Identity: 99.93%), and a IncX1 pN19335 plasmid that is closest to *Yokenella regensburgei* W13 plasmid pYRW13-125 (Genbank accession CP050812.1) (Zhou et al., 2020) (Coverage: 61%, Identity: 99.82%). Both plasmids only have a fragment homologous to their, respectively, most similar known plasmids (Figures 5D,E). pN19332 is a mobile plasmid encoding the type IV secretion system, the same as pA382_p2. Both pN19332 and pA382_p2 share the same backbone, whereas pN19332 also encodes an extra type II VapBC toxin-antitoxin system, and a *β*-lactam resistance gene *bla*_CTX-M-35_ gene. On the contrary, pN19335 and pYRW13-125 do not share the plasmid backbone that encodes key plasmid replication and mainteinance proteins, but share a large chunk of functional DNA.

**Figure 6:**
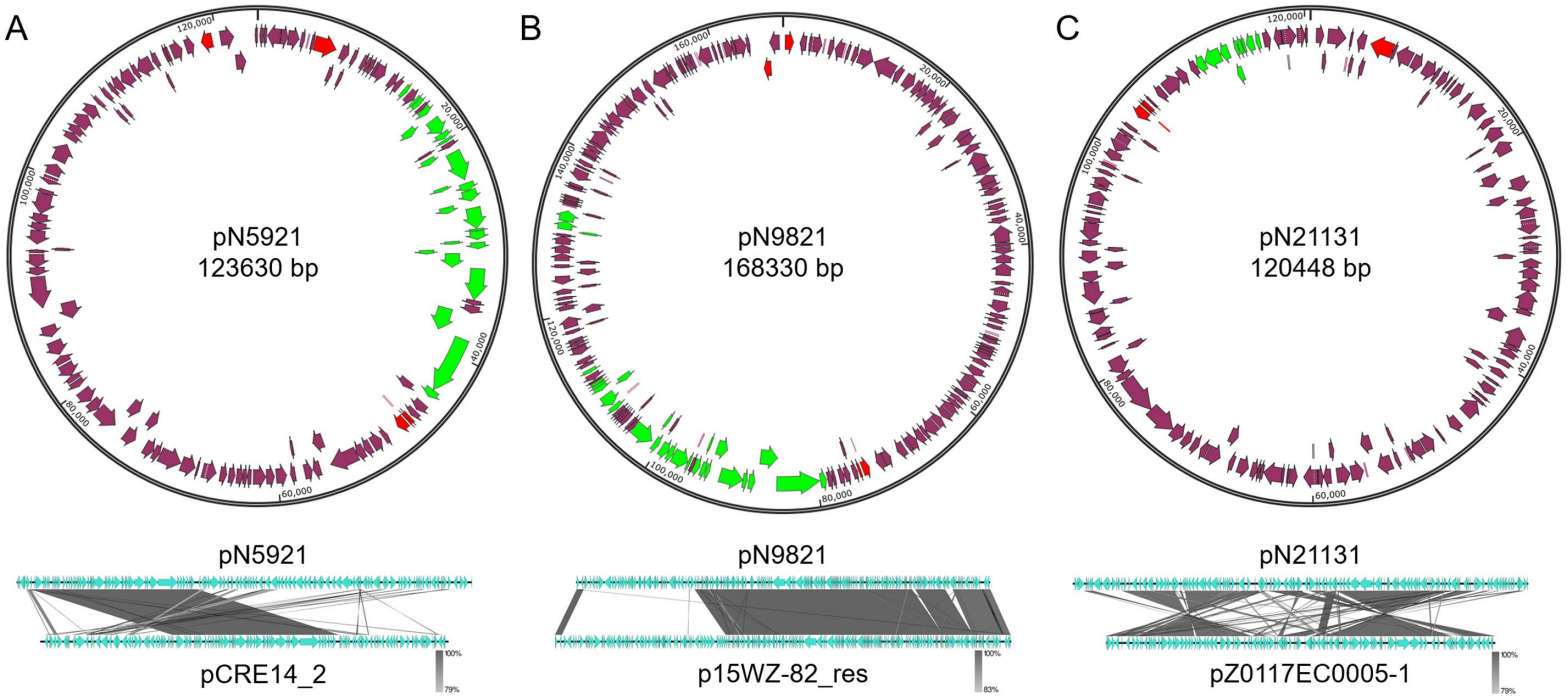
Structures of pN5921, pN9821, and pN21131. **(A)** pN5921; **(B)** pN9821; **(C)** pN21131. Green color indicates mobility related genes. Red color indicates the *rep* gene. Purple arrows indicate coding regions.

The three newly identified large plasmids (>100 kb) are mobile multidrug resistant plasmids that encode type IV secretion systems (Figure 6). All of them encode multiple Rep proteins, suggesting multiple recombination/integration events took place leading to these complex plasmids. All of the three plasmids are IncF plasmids.

*C. portucalensis* N59-2 carries a 123.6 kb pN5921 plasmid that shows closest homology with the *K. pneumoniae* S14_CRE14 plasmid pCRE14_2 (Genbank accession CP074530.1)^5^ (Coverage: 46%, Identity: 99.81%). The two plasmids share a backbone with key plasmid maintenance and mobilization genes, but differ in more than 50% of the DNA that encode functional genes (Figure 6A). These genes include sugar transport gene clusters, and interestingly, a polymyxin resistance gene *mcr-10.1* gene.

*K.* var*iicolo* N98-2 carry a 168.3 kb pN9821 plasmid that is closely related to p15WZ-82_res from *K. variicola* 15WZ-82 (Genbank accession CP032357.1)^6^ (Coverage: 70%, Identity: 99.94%). Similarly to the pN5921/pCRE14_2 plasmid pair, pN9821 and p15WZ-82_res share the same plasmid backbone, and differ in about a third of the genes (Figure 6B).

*E. coli* N211-3 carries a 120.4 kb pN21131 plasmid that appears to be similar to *E. coli* strain Z0117EC0005 plasmid pZ0117EC0005-1 (Genbank CP098224.1)^7^ (Coverage: 77%, Identity: 98.74%). The two plasmids share a large portion of DNA, but two inversion events took place during the evolution of the two plasmids (Figure 6C). In addition, pN21131 gained the type IV secretion system gene cluster over pZ0117EC0005-1, making it a mobile plasmid.

The surprising diversity of new plasmids found in this work confirms our lack of knowledge on plasmids, which are versatile and the most active parts of bacterial genome. They carry and transmit function across cell barriers, and modify bacterial function in a quick and adaptive manner. This is echoed by the finding that about half of the newly identified plasmids are mobile. The identification of so many new plasmids in one attempt using 3rd generation Nanopore sequencing confirms long-read high-throughput sequencing methods as powerful tools in plasmid discovery and surveillance.

**Figure 7:**
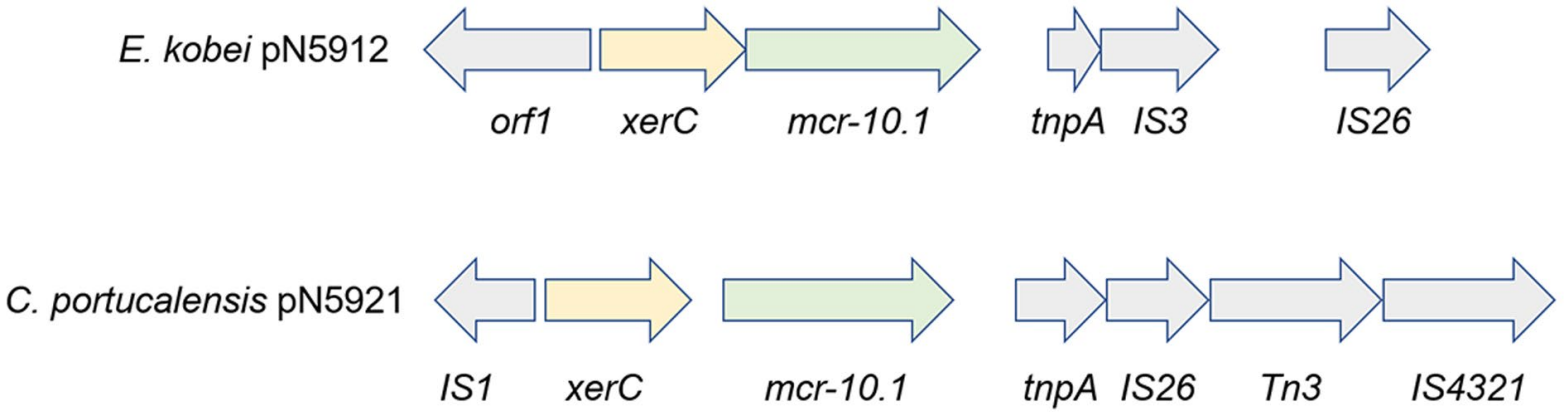
*mcr-10.1*-harboring gene clusters. Arrow lengths are proportional to gene sizes.

Several interesting findings can be made with the newly identified plasmids in this work. In comparison with their closest kin, some share the same backbone but acquire functional gene clusters, some retain functional gene clusters but have distinct backbones, some can acquire mobility gene clusters leading to increased mobility, and integration/recombination events can be clearly visualized during comparison.

One particular interesting finding is a new *mcr-10.1*-harboring mobile plasmid from *C. portucalensis* N59-2. This ARG confers resistance to polymyxin, an antibiotic of last resort the received tremendous attention in recent years (Liu et al., 2016). To date, some *mcr*-10.1 harboring plasmids have been reported (Guan et al., 2022; Kigen et al., 2024; Lei et al., 2020; Liu et al., 2022). These experimentally-verified plasmids were hosted with *E. cloacae* (Guan et al., 2022; Kigen et al., 2024), *E. roggenkampii* (Lei et al., 2020), and *Klebsiella* strains (Liu et al., 2022). No *mcr-10.1*-hosting *C. portucalensis* strains have been previously reported. This identification of a new, conjugation apparatus-coding, *mcr*-*10.1*-hosting *C. portucalensis* pN59-2 plasmid further expands our knowledge on the spectrum of *mcr-10.1* hosts.

### 3.4 Antimicrobial resistance of isolated strains

The presence of ARGs in isolated strains was investigated with AMRFinder (Table 2). Of all strains sequenced, only one *E. coli* strain did not carry any ARGs. Most of the ARGs are plasmid-borne, suggesting the extensive prevalence of ARGs and AMR.

**Table 2:** Antimicrobial resistance genes.

Strain	Taxonomy	Chromosomal ARG	Plasmid ARGs
N6-1	*Enterococcus faecalis*	*lsaA*	*tetM, tetL, aph(3′)-IIIa, sat4, ant(6)-Ia, lnuB, lsaE, spw, ant(6)-Ia, aac(6′)-Ie/aph(2″)-Ia, ermB*
N6-2	*Enterococcus faecalis*	*lsaA*	*tetM, tetL, aph(3′)-IIIa, sat4, ant(6)-Ia, lnuB, lsaE, spw, ant(6)-Ia, aac(6′)-Ie/aph(2″)-Ia, ermB, tetM, tetL, ermB, ant(6)-Ia, spw, lsaE, lnuB, ant(6)-Ia, sat4, aph(3′)-IIIa*
N6-3	*Enterococcus faecalis*	*lsaA*	*tetM, tetL, aph(3′)-IIIa, sat4, ant(6)-Ia, lnuB, lsaE, spw, ant(6)-Ia, aac(6′)-Ie/aph(2″)-Ia, ermB*
N59-1	*Enterobacter kobei*	*oqxA, oqxB, bla*_ACT-52_	*mcr-10.1*
N59-2	*Citrobacter portucalensis*	*bla*_CMY_*, qnrB9*	*mcr-10.1*
N59-3	*Escherichia coli*		*bla*_TEM-1_, *aac(3)-IId, mphA, sul1, qacEΔ1, aadA5, dfrA17*
N98-1	*Klebsiella quasipneumoniae*	*bla*_OKP-B-5_, *oqxA, oqxB, fosA*	–
N98-2	*Klebsiella variicola*	*bla*_LEN-2_, *fosA, oqxA, oqxB15*	*bla*_OXA-926_
N98-3	*Escherichia coli*	-	–
N112-1	*Staphylococcus aureus*	*blaZ*, *blaI*, *tet38, blaZ, blaI*	–
N112-2	*Escherichia coli*	*bla*_CTX-M-24_, *bla*_TEM-1_, *aac(3)-IId*, *mphA, sul1, qacEΔ1*, *aadA5, dfrA17*	–
N112-3	*Staphylococcus aureus*	*blaZ*, *blaI*, *tet38*	–
N183-1	*Klebsiella pneumoniae*	*qnrS1*, *dfrA14*, *floR*, *tetD*, *bla*_SHV-2_	*bla*_SHV-1_, *oqxA*, *oqxB32*, *fosA*
N183-2	*Escherichia coli*	–	*bla*_TEM-1_, *tetA, aph(6)-Id, aph(3″)-Ib, sul2, mphA, sul1, qacEΔ1, aadA5, dfrA17*
N183-3	*Escherichia coli*	–	*bla*_CTX-M-65_, *floR, tetA, dfrA14, aadA1, bla*_OXA-10_*, cmlA5, aar-2, qnrS1, tetA, bla*_TEM-1_, *aadA22, lnuF, aph(3″)-Ib, aph(6)-Id, aph(3′)-Ia, aph(4)-Ia, aac(3)-IVa*
N119-1	*Klebsiella pneumoniae*	*bla*_SHV-11_, *oqxA, oqxB19, fosA*	
N119-2	*Escherichia coli*	*bla*_CMY-2_, *bla*_CMY-2_	*aph(3′)-IIa, ble, bla*_TEM-1_, *sul2, aph(3″)-Ib, aph(6)-Id, tetA, floR, aac(3)-IId*
N119-3	*Klebsiella pneumoniae*	*bla*_SHV-11_, *oqxA*, *oqxB19, fosA*	
N129-1	*Proteus mirabilis*	*catA, tetJ*	
N129-2	*Proteus mirabilis*	*catA, tetJ*	
N129-3	*Escherichia coli*		*tetA, aph(6)-Id, aph(3″)-Ib, sul2, bla*_TEM-1_, *aac(3)-IID, dfrA17*
N157-1	*Klebsiella pneumoniae*	*bla*_SHV-11_, *oqxA*, *oqxB, fosA*	*tetD, sul1, qacEΔ1, qnrB6, sul1, qacEΔ1, aadA16, dfrA27, aar-3, aac(6′)-Ib-cr5, aac(3)-IId, bla*_TEM-1_, *tetA, floR, sul2, bla*_SHV-2_
N157-2	*Escherichia coli*	*tetA*	*bla*_CTX-M-65_, *aac(3)-IVa, aph(4)-Ia, sul2, floR, bla*_TEM-1_, *mphA, sul1, qacEΔ1, aadA5, dfrA17*
N157-3	*Klebsiella pneumoniae*	*bla*_SHV-11_, *oqxA*, *oqxB, fosA*	*tetD, sul1, qacEΔ1, qnrB6, sul1, qacEΔ1, aadA16, dfrA27, aar-3, aac(6′)-Ib-cr5, aac(3)-IId, bla*_TEM-1_, *tetA, floR, sul2, bla*_SHV-2_
N176-1	*Enterobacter hormaechei*	*bla*_ACT-46_, *oqxA*, *oqxB, fosA*	
N176-2	*Enterobacter hormaechei*	*bla*_ACT-46_, *oqxA*, *oqxB, fosA*	
N176-3	*Enterobacter hormaechei*	*bla*_ACT-46_, *oqxA*, *oqxB, fosA*	
N193-1	*Escherichia coli*	*sul1, qacEΔ1, aadA5, dfrA17, bla*_CTX-M-15_, *catB3, bla*_OXA-1_, *aac(6′)-Ib-cr5, mphA, tetB*	
N193-2	*Escherichia coli*		*mphA*
N193-3	*Escherichia coli*		*bla*_CTX-M-55_, *bla*_CTX-M-55_, *qnrS1*, *tetA*, *aph(3″)-IIa, ble, oqxB, oqxA2, sul2, floR, dfrA14, aadA1, bla*_OXA-10_, *cmlA5, aar-2*
N211-1	*Klebsiella pneumoniae*	*bla*_SHV-11_, *oqxA*, *oqxB19, fosA*	
N211-2	*Klebsiella pneumoniae*	*bla*_SHV-11_, *oqxA*, *oqxB19, fosA*	
N211-3	*Escherichia coli*		*bla*_TEM_, *mphA, sul1, qacE, bla*_DHA-1_, *qnrB4*

A total of nine integrons were found in seven strains, including two strains carrying two integrons on the plasmid. Two integrons are located on the chromosome, and all the rest are located on plasmids. One interesting integron carried by the *K. pneumoniae* IncFIA/IncR plasmid pN15712 has a gene cassette array *aac(3)-IId - aac(6′)-Ib-cr5 - aar-3 - dfrA27 - aadA16 - qacEΔ1 - sul1*. It is an unusually large (7 genes), multidrug resistant (aminoglycoside, fluoroquinolone, rifampicin, trimethoprim, quaternary ammonium, and sulfonamide) integron.

An additional interesting finding is the presence of polymyxin resistance-conferring *mcr-10.1* on plasmids. In addition to the just reported presence on pN5921 in *C. portucalensis* N59-2, it is also present on a second conjugative plasmid pN5912 in *E. kobei* pN5912 (Figure 7). To the best of our knowledge, *mcr-10.1* has not been reported in *C. portucalensis*. Comparison with the gene cluster surrounding *mcr-10.1* in pN5912 and pN5921 showed that, except for *xerC* that is conserved in all *mcr-10.1* containing clusters, the gene cluster organizations are significantly different. This suggest that the two conjugative *mcr-10.1*-carrying plasmids do not have the same *mcr-10.1* origin, and their co-occurrence in the same subject was not due to simple horizontal gene transfer. This may warn us that *mcr-10.1*-conferring polymyxin resistance may have already been quite prevalent.

### 3.5 Identical strains in abscess and skin implies a possible skin-origin infection

One thing that came to our attention while analyzing the isolated strains is the finding of two *S. aureus* strains. The two strains were isolated from the anal skin and abscess of patient N112. They harbor identical plasmids (pN11211), and compariative analysis showed that they only differed by 13 SNPs. Considering after assembly, an error rate of 0.01% is still highly possible, we came to the conclusion that the two strains, *S. aureus* N112-1 from anal skin and *S. aureus* N112-3 from abscess are identical strains. *Staphylococcus* is a common skin bacterium and rare in the gastrointestinal tract. Therefore, we believe this finding implies that the bacterium that is leading to infection and present in perianal abscess for patient N112 has a skin origin. This finding suggests the possibility that perianal abscess can be caused by skin microbes.

## 4 Conclusion

In this work, the high quality, 3rd generation sequencing-based, near perfect whole genome sequences of 33 bacterial strains from 11 perianal abscess-suffering patients were studied, particularly focusing on their plasmid diversity. A surprisingly high proportion of new plasmids were found, with the identification of 12 new plasmids that have not been observed before, possibly because of the power of long-read sequencing that made high-accuracy discovery and surveillance of plasmids possible. These new plasmids, when compared with their closest homologs, showed significant differences, which further confirms the strong dynamics of plasmids, the key to genomic plasticity. Of particular interest, two polymyxin-resistance-conferring, *mcr*-*10.1*-carrying conjugative plasmids were found in *C. portucalensis* and *E. kobei* strains. This is the first finding of *mcr-10.1* in *C. portucalensis*, and one of the first few evidences of conjugative, mobile *mcr-10.1*-carrying plasmids. This work shows the strengths of long-read sequencing on identification of plasmids, our lack of knowledge on plasmids, and diversity of plasmid structures.

## Data Availability

The datasets presented in this study can be found in online repositories. The names of the repository/repositories and accession number(s) can be found at: https://www.ncbi.nlm.nih.gov/genbank/, PRJNA1120246.

## Footnotes

^1^ https://github.com/nanoporetech/dorado/

^2^ https://github.com/tseemann/snippy

^3^ https://www.ncbi.nlm.nih.gov/nuccore/CP044388.1

^4^ https://www.ncbi.nlm.nih.gov/nuccore/OR095745.1

^5^ https://www.ncbi.nlm.nih.gov/nuccore/CP074530.1

^6^ https://www.ncbi.nlm.nih.gov/nuccore/CP032357.1

^7^ https://www.ncbi.nlm.nih.gov/nuccore/CP098224.1

## References

[ref1] Afşarlar C. E. Karaman A. Tanır G. Karaman I. Yılmaz E. Erdoğan D.. (2011). Perianal abscess and fistula-in-ano in children: clinical characteristic, management and outcome. Pediatr. Surg. Int. 27, 1063–1068. doi: 10.1007/s00383-011-2956-7, PMID: 21785979

[ref2] Arredondo-Alonso S. Willems R. J. van Schaik W. Schürch A. C. (2017). On the (im)possibility of reconstructing plasmids from whole-genome short-read sequencing data. Microb. Genom. 3:e000128. doi: 10.1099/mgen.0.000128, PMID: 29177087 PMC5695206

[ref3] Brar M. S. Remzi F. Warusavitarne J. Datta I. (2020). Does antibiotic therapy prevent fistula in-ano after incision and drainage of simple perianal abscess? Canadian journal of surgery. J. Can. Chirurgie 63, E362–E364. doi: 10.1503/cjs.017119, PMID: 32813483 PMC7458676

[ref4] Camacho C. Coulouris G. Avagyan V. Ma N. Papadopoulos J. Bealer K.. (2009). BLAST+: architecture and applications. BMC Bioinf. 10:421. doi: 10.1186/1471-2105-10-421, PMID: 20003500 PMC2803857

[ref5] Carattoli A. Hasman H. (2020). Plasmid finder and in silico pMLST: identification and typing of plasmid replicons in whole-genome sequencing (WGS). Methods Mol. Biol. 2075, 285–294. doi: 10.1007/978-1-4939-9877-7_20, PMID: 31584170

[ref6] Carroll A. C. Wong A. (2018). Plasmid persistence: costs, benefits, and the plasmid paradox. Can. J. Microbiol. 64, 293–304. doi: 10.1139/cjm-2017-0609, PMID: 29562144

[ref7] Chaumeil P.-A. Mussig A. J. Hugenholtz P. Parks D. H. (2022). GTDB-Tk v2: memory friendly classification with the genome taxonomy database. Bioinformatics 38, 5315–5316. doi: 10.1093/bioinformatics/btac672, PMID: 36218463 PMC9710552

[ref8] Chen W. Wang Q. Wu H. Xia P. Tian R. Li R.. (2024). Molecular epidemiology, phenotypic and genomic characterization of antibiotic-resistant enterococcal isolates from diverse farm animals in Xinjiang, China. Sci. Total Environ. 912:168683. doi: 10.1016/j.scitotenv.2023.168683, PMID: 37996027

[ref9] De Coster W. Rademakers R. (2023). NanoPack2: population-scale evaluation of long-read sequencing data. Bioinformatics 39:btad311. doi: 10.1093/bioinformatics/btad311, PMID: 37171891 PMC10196664

[ref10] Feldgarden M. Brover V. Gonzalez-Escalona N. Frye J. G. Haendiges J. Haft D. H.. (2021). AMRFinderPlus and the reference gene catalog facilitate examination of the genomic links among antimicrobial resistance, stress response, and virulence. Sci. Rep. 11:12728. doi: 10.1038/s41598-021-91456-0, PMID: 34135355 PMC8208984

[ref11] Ghahramani L. Minaie M. R. Arasteh P. Hosseini S. V. Izadpanah A. Bananzadeh A. M.. (2017). Antibiotic therapy for prevention of fistula in-ano after incision and drainage of simple perianal abscess: a randomized single blind clinical trial. Surgery 162, 1017–1025. doi: 10.1016/j.surg.2017.07.001, PMID: 28822559

[ref12] Green E. D. Watson J. D. Collins F. S. (2015). Human genome project: twenty-five years of big biology. Nature 526, 29–31. doi: 10.1038/526029a, PMID: 26432225 PMC5101944

[ref13] Guan J. Li L. Zheng L. Lu G. Wang Y. Lakoh S.. (2022). First report of the colistin resistance gene mcr-10.1 carried by IncpA1763-KPC plasmid pSL12517-mcr10.1 in *Enterobacter cloacae* in Sierra Leone. Microbiol. Spectr. 10:e0112722. doi: 10.1128/spectrum.01127-22, PMID: 35695522 PMC9431528

[ref14] Hu T. Chitnis N. Monos D. Dinh A. (2021). Next-generation sequencing technologies: an overview. Hum. Immunol. 82, 801–811. doi: 10.1016/j.humimm.2021.02.012 33745759

[ref15] Kigen C. Muraya A. Wachira J. Musila L. (2024). The first report of the mobile colistin resistance gene, mcr-10.1, in Kenya and a novel mutation in the phoQ gene (S244T) in a colistin-resistant *Enterobacter cloacae* clinical isolate. Microbiol. Spectr. 12:e0185523. doi: 10.1128/spectrum.01855-23, PMID: 38230935 PMC10846102

[ref16] Kolmogorov M. Yuan J. Lin Y. Pevzner P. A. (2019). Assembly of long, error-prone reads using repeat graphs. Nat. Biotechnol. 37, 540–546. doi: 10.1038/s41587-019-0072-8, PMID: 30936562

[ref17] Lei C.-W. Zhang Y. Wang Y.-T. Wang H.-N. (2020). Detection of mobile colistin resistance gene mcr-10.1 in a conjugative plasmid from *Enterobacter roggenkampii* of chicken origin in China. Antimicrob. Agents Chemother. 64, e01191–e01120. doi: 10.1128/AAC.01191-20, PMID: 32718964 PMC7508621

[ref18] Li X. Hu H. Zhu Y. Wang T. Lu Y. Wang X.. (2024). Population structure and antibiotic resistance of swine extraintestinal pathogenic *Escherichia coli* from China. Nat. Commun. 15:5811. doi: 10.1038/s41467-024-50268-2, PMID: 38987310 PMC11237156

[ref19] Li W. O’Neill K. R. Haft D. H. DiCuccio M. Chetvernin V. Badretdin A.. (2021). RefSeq: expanding the prokaryotic genome annotation pipeline reach with protein family model curation. Nucleic Acids Res. 49, D1020–D1028. doi: 10.1093/nar/gkaa1105, PMID: 33270901 PMC7779008

[ref20] Li L. Yu T. Ma Y. Yang Z. Wang W. Song X.. (2018). The genetic structures of an extensively drug resistant (XDR) *Klebsiella pneumoniae* and its plasmids. Front. Cell. Infect. Microbiol. 8:446. doi: 10.3389/fcimb.2018.00446, PMID: 30662878 PMC6328971

[ref21] Liu M. C. Jian Z. Liu W. Li J. Pei N. (2022). One health analysis of mcr-carrying plasmids and emergence of mcr-10.1 in three species of *Klebsiella* recovered from humans in China. Microbiol. Spectr. 10:e0230622. doi: 10.1128/spectrum.02306-22, PMID: 36287001 PMC9769640

[ref22] Liu Y.-Y. Wang Y. Walsh T. R. Yi L.-X. Zhang R. Spencer J.. (2016). Emergence of plasmid-mediated colistin resistance mechanism MCR-1 in animals and human beings in China: a microbiological and molecular biological study. Lancet Infect. Dis. 16, 161–168. doi: 10.1016/S1473-3099(15)00424-7 26603172

[ref23] Lohsiriwat V. Yodying H. Lohsiriwat D. (2010). Incidence and factors influencing the development of fistula-in-ano after incision and drainage of perianal abscesses. J. Med. Assoc. Thailand 93, 61–65, PMID: 20196412

[ref24] Loman N. J. Quick J. Simpson J. T. (2015). A complete bacterial genome assembled de novo using only nanopore sequencing data. Nat. Methods 12, 733–735. doi: 10.1038/nmeth.3444, PMID: 26076426

[ref25] Manni M. Berkeley M. R. Seppey M. Simão F. A. Zdobnov E. M. (2021). BUSCO update: novel and streamlined workflows along with broader and deeper phylogenetic coverage for scoring of eukaryotic, prokaryotic, and viral genomes. Mol. Biol. Evol. 38, 4647–4654. doi: 10.1093/molbev/msab199, PMID: 34320186 PMC8476166

[ref26] Mikheenko A. Prjibelski A. Saveliev V. Antipov D. Gurevich A. (2018). Versatile genome assembly evaluation with QUAST-LG. Bioinformatics 34, i142–i150. doi: 10.1093/bioinformatics/bty266, PMID: 29949969 PMC6022658

[ref27] Mocanu V. Dang J. T. Ladak F. Tian C. Wang H. Birch D. W.. (2019). Antibiotic use in prevention of anal fistulas following incision and drainage of anorectal abscesses: a systematic review and meta-analysis. Am. J. Surg. 217, 910–917. doi: 10.1016/j.amjsurg.2019.01.015, PMID: 30773213

[ref28] Parks D. H. Imelfort M. Skennerton C. T. Hugenholtz P. Tyson G. W. (2015). CheckM: assessing the quality of microbial genomes recovered from isolates, single cells, and metagenomes. Genome Res. 25, 1043–1055. doi: 10.1101/gr.186072.114, PMID: 25977477 PMC4484387

[ref29] Partridge S. R. Kwong S. M. Firth N. Jensen S. O. (2018). Mobile genetic elements associated with antimicrobial resistance. Clin. Microbiol. Rev. 31, e00088–e00017. doi: 10.1128/CMR.00088-17, PMID: 30068738 PMC6148190

[ref30] Rozwandowicz M. Brouwer M. S. M. Fischer J. Wagenaar J. A. Gonzalez-Zorn B. Guerra B.. (2018). Plasmids carrying antimicrobial resistance genes in Enterobacteriaceae. J. Antimicrob. Chemother. 73, 1121–1137. doi: 10.1093/jac/dkx488 29370371

[ref31] Sahnan K. Adegbola S. O. Tozer P. J. Watfah J. Phillips R. K. (2017). Perianal abscess. BMJ 356:j475. doi: 10.1136/bmj.j475 28223268

[ref32] Seemann T. (2014). Prokka: rapid prokaryotic genome annotation. Bioinformatics 30, 2068–2069. doi: 10.1093/bioinformatics/btu153 24642063

[ref33] Song Y. (2021). Central dogma, redefined. Nat. Chem. Biol. 17:839. doi: 10.1038/s41589-021-00850-2, PMID: 34312560

[ref34] Sullivan M. J. Petty N. K. Beatson S. A. (2011). Easyfig: a genome comparison visualizer. Bioinformatics 27, 1009–1010. doi: 10.1093/bioinformatics/btr039, PMID: 21278367 PMC3065679

[ref35] Yin Z. Xia D. Shen M. Zhu D. Cai H. Wu M.. (2020). Tetracycline degradation by *Klebsiella* sp. strain TR5: proposed degradation pathway and possible genes involved. Chemosphere 253:126729. doi: 10.1016/j.chemosphere.2020.126729, PMID: 32289610

[ref36] Zhou D. Sun Z. Lu J. Liu H. Lu W. Lin H.. (2020). Characterization of a novel chromosomal class C β-lactamase, YOC-1, and comparative genomics analysis of a multidrug resistance plasmid in *Yokenella regensburgei* W13. Front. Microbiol. 11:2021. doi: 10.3389/fmicb.2020.02021, PMID: 32973731 PMC7468467

